# The cost of implementing measles campaign in Nigeria: comparing the stand-alone and the integrated strategy

**DOI:** 10.1186/s13561-023-00441-y

**Published:** 2023-06-13

**Authors:** Anne Eudes Jean Baptiste, Jurjen Van der Schans, Samuel Bawa, Balcha Masresha, John Wagai, Joseph Oteri, Boubacar Dieng, Margaret Soyemi, Rufus Eshuchi, Yared G. Yehualashet, Oluwole Afolabi, Fiona Braka, André Bita, Eelko Hak

**Affiliations:** 1 World Health Organization, Country Office, Abuja, Nigeria; 2 Department of Economics, Econometrics and Finance, Faculty of Economics & Business, University of Groningen, Groningen, The Netherlands; 3 World Health Organization, African Regional Office, Brazzaville, Congo; 4 National Primary Health Care Development Agency, Abuja, Nigeria; 5 Technical Assistance Consultant, Global Alliance for Vaccines and Immunizations (GAVI), Abuja, Nigeria; 6 United Nations Children’s Fund (UNICEF) - Country Office for Nigeria, Abuja, Nigeria; 7 Groningen Research Institute of Pharmacy, University of Groningen, Groningen, The Netherlands

**Keywords:** Cost-minimization, Measles, Supplementary immunization activities, Integration, Vaccination

## Abstract

**Background:**

Effective integration, one of the seven strategic priorities of the Immunization Agenda 2030, can contribute to increasing vaccination coverage and efficiency. The objective of the study is to measure and compare input costs of “non-selective” measles vaccination campaign as a stand-alone strategy and when integrated with another vaccination campaign.

**Methods:**

We conducted a cost-minimization study using a matched design and data from five states of Nigeria. We carried-out our analysis in 3 states that integrated measles vaccination with Meningitis A and the 2 states that implemented a stand-alone measles campaign. The operational costs (e.g., costs of personnel, training, supervision etc.) were extracted from the budgeted costs, the financial and technical reports. We further used the results of the coverage surveys to demonstrate that the strategies have similar health outputs.

**Results:**

The analysis of the impact on campaign budget (currency year: 2019) estimated that savings were up to 420,000 United States Dollar (USD) with the integrated strategies; Over 200 USD per 1,000 children in the target population for measles vaccination (0.2 USD per children) was saved in the studied states. The savings on the coverage survey components were accrued by lower costs in the integration of trainings, and through reduced field work and quality assurance measures costs.

**Conclusions:**

Integration translated to greater value in improving access and efficiency, as through sharing of costs, more life-saving interventions are made accessible to the communities. Important considerations for integration are resource needs, micro-planning adjustments, and health systems delivery platforms.

## Background

In 2011, the World Health Organization (WHO) African Region adopted a target of measles elimination by 2020 [1]. Despite significant progress in reducing measles burden and mortality, the disease is still common in many parts of the Region. By September 2020, only 13 (out of 47) countries had a measles incidence of less than 1 case/million population [2]. Consequently, the African Region has missed the 2020 elimination goal for measles.

In 2019, Nigeria has experienced repeated outbreaks of measles: Data from the measles surveillance database showed that 35 states out of its 36 states and the Federal Capital Territory (FCT) experienced at least one measles outbreak. Further assessment of the database by Nigerian National Primary Health Care Development Agency (NPHCDA) predicted massive measles outbreaks to occur between January and April 2020 in absence of a supplemental immunization activities (SIAs) in the fourth quarter of 2019. As a result, and to increase population immunity to measles, a follow-up campaign [3] in the Northern region of Nigeria was scheduled. However, several funding gaps were identified. To bridge the gaps in funding, for an effective utilization of human resources, and more importantly, to prevent disease outbreaks, the country decided to implement an integrated measles and MenAfriVac® (Men-A) vaccination campaign. Meningococcal meningitis is observed worldwide but the highest burden of the disease is in the meningitis belt of sub-Saharan Africa that includes 26 Nigerian’s States [4]. Both *Neisseria meningitidis* serogroup A and measles remain a major public health challenge in Nigeria.

The country launched the SIAs to reach children with measles and Men-A vaccines in November 2019. Fifteen states and the FCT implemented an integrated measles and Men-A campaign, and two states, Yobe and Kano, implemented a stand-alone measles campaign.

Integration is one of the six guiding principles of the Global Vaccine Action Plan 2011–2020, and the Immunization Agenda 2030 (IA2030). An effective integration between immunization and other health programs can contribute to increasing vaccination coverage [5]. Additionally, integrated service delivery may help to increase efficiency as operational costs are shared across programmes [6]. This may be a potential way of reducing the cost associated with the implementation of vaccination campaigns [7–9]. While many studies support the decision to integrate other health interventions in measles SIAs [10–15], data on the integration of measles with other injectable vaccines during an SIA are very limited, with the exception of unpublished reports from Ethiopia, Republics of Niger and Chad and, a review of public health interventions integrated with the introduction of the MenAfriVac®. The review conducted in 7 countries of the African meningitis belt, indicated that opportunities of integration are reduction of missed opportunities of vaccination, and reduction of cases and burden of the diseases [16].

The objective of our study is to measure and compare the costs of measles stand-alone vaccination campaign strategy with costs of measles vaccination campaign integrated with another vaccination campaign (Men-A). Thus, the operational cost and coverage of different vaccination strategies could help make recommendations to facilitate financial planning for future campaigns in Nigeria and elsewhere, and aid the elimination of measles in the WHO regions (No WHO region has achieved and maintained measles elimination).

## Methods

### Matched study design and study setting

We conducted a cost-minimization study using a matched design, and data collected from five states implementing SIAs in which the uptake is relatively similar between the two vaccination strategies (e.g., measles vaccination in a stand-alone versus measles vaccination in an integrated campaign). The objective of a cost minimization is to minimize cost more than maximize output as the effectiveness is similar. Because we have good evidence of effectiveness of measles vaccines [17], we are assuming that the outcomes of the strategies are the same. We did not evaluate the health impacts of the different strategies, only the coverage achieved. We did not conduct any sensitivity analyses; There is uncertainty around the different cost categories, however, considering the outcome differences of the total stand-alone vs. integrated campaign, we do not expect the uncertainty to overlap between the two strategies. Applying uncertainty (of 10–20% for example) would be arbitrary. Because our study only has two data points per cost category per strategy, uncertainty cannot be calculated. .

The matching was performed in a qualitative way, linking the selected states with an integrated campaign to the states with a stand-alone campaign of the measles vaccination, based on the most similar state in terms of location, population, and ratio of urban vs. rural: Our analysis was carried-out in 3 states that integrated measles vaccination matched with the 2 states that implemented the stand-alone measles campaigns. Thus, to compare the vaccination strategies, we matched Yobe (measles stand-alone) with Plateau State (measles integrated campaign) and, Kano State (measles stand-alone) with Kaduna and Kwara States (measles integrated campaign). Kano, historically, is one of the most difficult states to achieve high coverage. Kano conducted a measles stand-alone campaign as full national attention/support on the State was needed to maximize the quality of the SIA. With the remaining Men A doses, in 2018, a mini catch-up campaign was implemented in the Yobe state targeting children 1–6 years who were born after the mass vaccination of 2012. Therefore, Yobe was not due for Men A and implemented a measles stand-alone campaign. The selection to either perform either a stand-alone or integrated campaign was not based on previous performance. All the assessed states are from the Northern region of Nigeria where measles routine coverage is sub-optimal [18, 19]. We also reviewed the micro-plans and the team’s daily implementation plans (DIPs) of the matched states. Key considerations were: (1) No eligible person traveled more than 1 km and, (2) where necessary, the vaccination team moved from one vaccination post to another to reduce the walking distance of the caregivers. Therefore, we did not need to consider the surface area of the states and the average time spent by a caregiver to travel to and from the vaccination in our matching. We further used the results of the coverage surveys to demonstrate that the strategies have about the same coverage (Table 1). The cost-minimization analysis focused on the operational costs of the measles vaccination campaigns.

**Table 1:** Children vaccinated during measles and meningitis* supplemental immunization activities, Nigeria, 2019

		Intervention	Measles	Meningitis A
		Target age group	9–59 months	12–59 months
**Vaccination Strategy, Stand-Alone**	**Yobe**	Number vaccinated	870,946	
		Coverage by survey (%)	95.1	
		95% CI (%)	(90.7, 97.5)	
	**Kano**	Number vaccinated	2,790,912	
		Coverage by survey (%)	89.7	
		95% CI (%)	(86.2, 92.3)	
**Vaccination Strategy, Integration**	**Plateau**	Number vaccinated	958,846	900,386
		Coverage by survey (%)	94.0	93.4
		95% CI (%)	(89.7, 96.6)	(88.5, 96.3)
	**Kaduna**	Number vaccinated	2,439,351	1,948,185
		Coverage by survey (%)	91.2	90.3
		95% CI (%)	(84.2, 95.3)	(82.3, 94.9)
	**Kwara**	Number vaccinated	488,102	466,687
		Coverage by survey (%)	95.9	95.4
		95% CI (%)	(91.5, 98.1)	(89.6, 98.1)

* All children that received Meningitis A received measles. Children 9 to 11 months only received measles

Source: Vaccination team’s tally sheets and Nigeria National Bureau of Statistics (NBS) coverage survey reports

### Targeted study population characteristics

The 2019 measles vaccination campaign targeted over 22.2 million children from 9 to 59 months of age. Following the WHO guidelines on Men-A immunization and control, over 22.3 million children 1 to 5 years and 1 to 7 years were targeted during the Men-A catch-up campaign. As the meningitis mass campaigns were implemented in the country on a phased manner from 2011 to 2014, the catch-up campaigns were for birth cohorts born since the initial mass vaccination and which would not be within the age range targeted by the routine immunization programme [20].

A micro-plan process was conducted by the State PHCDA and led to the validation of the vaccination team’s workload in delivery of the vaccines. Subsequently, a verification process was carried out to assess the accuracy and consistency of the micro-plan in readiness for the campaign by reviewing documented campaign requirements. It is expected that the variance in target population should be realistic (less than 5% variance). The target age-group and the verified target population were based on the micro-plan verification exercise. Therefore, the estimated size of the verified targeted population of Kano was 2,940,912 children 9–59 months, which corresponds to the sum of the estimates for Kaduna and Kwara States (2,967,453 children). Similarly, the estimated size of the verified targeted population of Yobe State of 970,946 children 9–59 months is close to estimates for Plateau (1,099,512 children).

### Vaccination strategy and layout

The organization of the vaccination sites were the same for stand-alone and integrated strategies, and were either fixed or temporary sites. The fixed posts were in a Dispensary, Health Post, Primary Health Centre, clinic or a hospital. The temporary posts were set up in hard-to-reach areas and other places (where vaccination activities do not usually occur) using any suitable shelter, schools, mosques/churches, market areas. The location of the temporary posts could change daily, but this was indicated in the team’s DIPs.

The stand-alone measles vaccination campaigns were planned to cover 150 children per day per team, and lasted a total of 5 days, while the integrated measles-meningitis vaccination campaign vaccination was planned to provide an average of 300 doses of vaccine (including both antigens) over an 8-days period of implementation. Each vaccination team of the stand-alone campaigns comprised of 7 Persons (e.g., 2 vaccinators, 2 recorders, 1 crowd controller, 1 town announcer and 1 House-to-House mobiliser), while each vaccination team of the integrated measles and Men A campaigns comprised of 10 persons (e.g., 3 vaccinators, 3 recorders, 1 crowd controller, 1 screener at the screening point, 1 town announcer and 1 House-to-House mobiliser).

Advocacy, Communication and Social Mobilization (ACSM) activities were carried out for efficient awareness and demand creation for immunization and other health care services provided during the campaign. The ACSM comprised of community engagement/mobilization, individual and household engagement/mobilization, and health education. Every vaccination post was clearly marked (e.g. poster, banner, flag) and all team member were identified by either their armband, aprons, and T-shirt with an SIA sign.

### Vaccination coverage survey

**Fig. 1:**
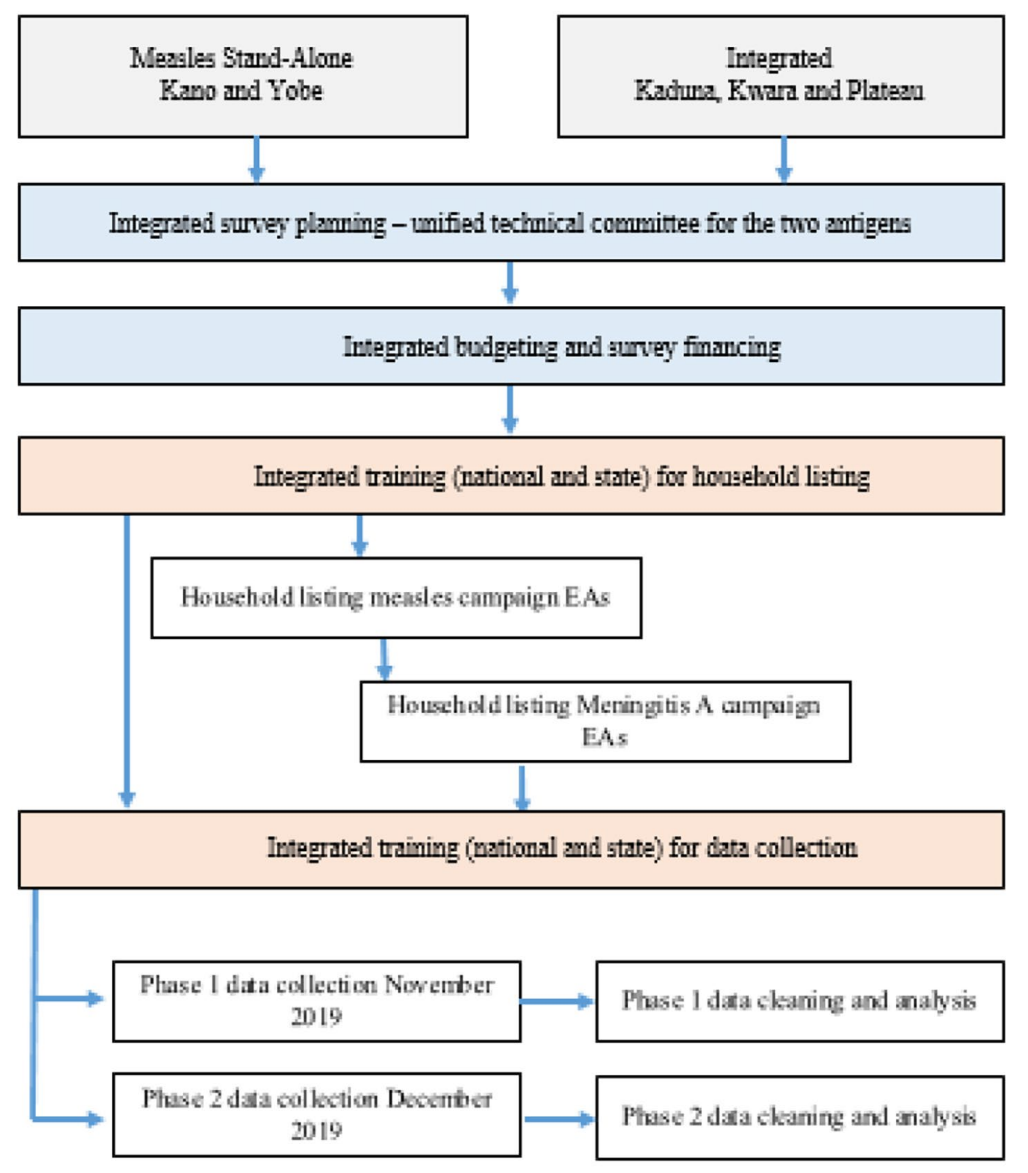
Schematic showing the integrated planning, integrated campaigns and data collection for coverage survey. EAs = Enumeration Areas

The post-vaccination campaign coverage survey was conducted following the SIAs between November 2019 and February 2020. The survey was a cross-sectional household-based survey conducted on a probability sample of 8,755 households in all 20 states in Northern Nigeria and in FCT-Abuja. The survey estimated and provided information on the children receiving vaccination during the campaign. In states where more than one vaccine antigen was administered (e.g., Plateau, Kwara and Kaduna) an integrated approach was adopted from the planning to the implementation of the survey. The integrated approach involved an integrated survey planning (e.g., unified technical committee for measles and Men-A), integrated budgeting and survey financing, integrated training (national and state) for household listing and, field work was conducted in phases based on the timing of campaign (Fig. 1).

### Information on cost estimates

We compared the service delivery costs associated with the implementation of a measles stand-alone and when integrated with another campaign in the respective states. Costs are therefore related to implementation of measles SIAs with fixed costs already separated between the vaccination campaigns. The operational costs were extracted from the budgeted costs following the micro-plan verification process where consent for the operational target population were reached. The budget was prepared using Gavi budget template, Gavi cost grouping and activity classification. Additional data were gathered from financial and technical reports showing the immunization activities implemented, amount disbursed, and proportion of budget covered. The major budget lines and definitions of the various cost items are provided in Table 2.

**Table 2:** Summary definitions of cost categories

Cost categories - Supplemental Immunization Activity		
campaign planning and preparation components costs		
	Unit	Definition
Social Mobilization		
	Production/distribution of IEC materials	Production of flyers, banners, FAQs etc. for information, education and communication
	Media engagements	Radio spots production and broadcast, documentation, production of 2 radio spots in 14 languages, airing of radio jingles etc.
	Stakeholder’s engagements	This is for engagement of State, LGA and Ward level stake holders using edutainment and other engagement activities at LGA level.
	Tracking and monitoring	Pre - Implementation tracking by Government personnel for monitoring pre-implementation activities
Training		
	Implementation trainings (State/LGA/Ward/Independent Monitors)	State/LGAs/Ward implementation trainings. Independent Monitors training is done a week prior to implementation
	Micro-Plan process	Microplanning Training of Trainers is done centrally for all stakeholders. The training is further conducted at the States. Ward level micro planning process is done with very high-level participation of the community
	Micro-Plan Verification	Verification of Micro-Plans from States by Government personnel
Admin.		
	Technical Assistance	Technical Assistance from WHO and UNICEF to strengthen the immunization program at State level
Overhead		
	Office operational costs	Provision is made per State and Zonal Offices as operational cost to take care of minor expenses in the office during implementation
Supervision		
	National Supervision	Required for facilitation of the Government personnel to supervise the campaign
**Vaccine delivery components costs**		
Vaccination personnel		
	Vaccination Personnel	Vaccination personnel and State Team members are engaged per State to work
	Transport allowance for collection of payment	Transport allowance to vaccination personnel to enable them travel to the designated payment site to collect their stipend
Implementation Materials		
	Implementation Materials - State Level	Implementation materials to be produced at the state level
Transport and logistics		
	Teams and supplies transport	Transportation of vaccination teams (team logistic). Transportation of materials from National to State levels during implementation
	Waste Management	Required to collect from facility and transported to wards in readiness for disposal
	Pen Markers	Required for finger marking of vaccinated children
**Cost categories - Post Campaign Coverage Survey**		
Trainings		
	Trainings	Training for enumerators and data collectors
Coverage survey		
	Fieldwork and Quality Assurance Measures	Data collection, monitoring and coordination
	Personnel	Data collectors, enumerators, training facilitators and supervisors

IEC = information, education, and communication; LGA = Local Government Area

### Cost estimations and unit costs

#### Included costs

The costing study include measles vaccination related costs from (1) campaign planning and preparation components, (2) vaccine delivery and (3) evaluation of the campaign. The studied costs were presented as the operational cost of the measles stand-alone, and measles integrated with another vaccination campaign (Men A) per unit and per 1,000 children in the target population for measles vaccination. The results are further broken down to show differences between states, vaccination strategy, as well as cost items and activities. The budget impact on stand-alone per 1,000 children per state was calculated by multiplying the cost differences (costs per 1,000 children in integrated minus stand-alone states) between matched states with the target population.

#### Excluded costs

There are costs that were excluded from our analysis. For example, costs specific to measles surveillance (e.g., sample transportation) during campaign were not considered since these costs are not associated with the conducting of campaigns. Likewise, related productivity losses associated with the average time spent by a caregiver/vaccinees to travel to and from the vaccination site (average wage) were not included in the analysis as the vaccinees did not travel more than 1 km according to the vaccination team’s DIPs. Social mobilization activities at central level (FCT) were also excluded as only minor adjustments to messaging was done for the integrated strategy. We did not consider implementation materials (e.g., field guide, manuals, data management form – tally sheets, vaccination cards, and other materials) to be produced at central level since it is linked to the target population and vaccination personnel, the output (cost per 1,000 children) will be like that of vaccination personnel.

The SIAs were funded primarily by GAVI, as per the budget that was developed and approved, and covered the cost of bundled vaccines, as well as most of the operational costs. The Federal Government of Nigeria was expected to mobilize the additional funds to cover the gaps in the operational costs but could not provide its contribution. To bridge the funding gaps, the country integrated the campaigns. Nevertheless, the State Government released some limited funds to support the Local Government Areas (LGAs or Districts) (e.g., State immunization officers’ supportive supervision, additional logistics to vaccination team working in hard-to-reach/far-to-reach communities). While the needed funds can be calculated, the amount released by the States was not always documented and was excluded from our analysis. Likewise, costs related with the procurement of adverse event following immunization (AEFI) kits were not documented.

## Results

The total number of children vaccinated according to the administrative reports are indicated in Table 1. The results of the survey showed that Kwara (integrated campaign) and Yobe State (stand-alone) vaccinated 95.9% (95%CI: 91.5–98.1) and 95.1% (95%CI: 90.7–97.5) of all children in the target age group with measles. The programmatic coverage threshold set for measles campaigns was 95% in all administrative units. Over 89.7% (95%CI: 86.2–92.3), 91.2% (95%CI: 84.2–95.3) and 94.0% (95%CI: 89.7–96.6) of eligible children for measles in Kano (stand-alone), Kaduna and Plateau (integrated campaigns) received measles vaccination during the campaigns respectively. All the states that have conducted Men-A SIAs achieved the 80% targeted coverage (Table 1). The 95% CIs of the coverage rates are largely overlapping hence there is no statistically significant difference in vaccine uptake.

A comparison of the actual costs with the budgeted costs for the campaigns has shown no significant budget variances. For example, less than 3,200 United States Dollar (USD) variance was found between the actual and the budgeted costs for Kaduna State, around 1,300 USD between the actual cost and budgeted costs in Kwara, and 1,246 USD between the actual and budgeted cost in Plateau State. The results are in conformance with those for other states.

The campaign planning and preparation cost for the stand-alone vaccination campaign in Yobe (stand-alone) was 0.137 USD per children, whereas the cost for the integrated campaigns in Plateau State (integrated) was 0.184 USD per children. Similarly, the stand-alone vaccination campaign in the state of Kano had a cost of 0.095 USD per children while its matched (Kaduna and Kwara) states had a cost of 0.133 USD per children.

The costs associated with communication materials and the community engagements in Yobe and Kano (stand-alone) totaled 48,481 USD and 133,224 USD, compared to 110,697 USD and 196,867 USD in their respective matched states. Total amounts for implementation trainings were 11,131 USD and 29,034 USD in Yobe and Kano, compared to 22,738 USD and 55,194 USD in their respectively matched integrated campaigns states. However, major cost-savings were achieved through the micro-plan process as it was integrated and, to a much lesser extent, during stakeholders (State/LGA) and media engagements, technical assistance, and office operational costs for only the Yobe and Plateau comparison. (Table 3).

**Table 3:** Campaign planning and preparation costs* by vaccination strategy, Nigeria, 2019

Cost categories	Measles target population (9–59 months)	Stand-Alone	Integrated	Stand-Alone	Integrated	Integrated	Integrated
		Yobe	Plateau	Kano	Kaduna	Kwara	Kaduna + Kwara
		970,946	1,099,512	2,940,912	2,439,351	528,102	2,967,453
**Social Mobilization**							
	Production/distribution of IEC materials	$ 8,940	$ 38,501	$ 25,707	$ 70,720	$ 26,405	$ 97,125
	Number of teams	1,295	458	3,921	1,016	220	1,236
	Costs per production/distribution of IEC material per teams	$ 7	$ 84	$ 7	$ 70	$ 120	$ 79
	Costs per 1,000 targeted children	$ 9.21	$ 35.02	$ 8.74	$ 28.99	$ 50.00	$ 32.73
	Number of production/distributions of IEC materials per 100,000 targeted children	133	42	133	42	42	42
	Stakeholder’s engagements (State/LGA/Ward)	$ 63,404	$ 93,348	$ 154,996	$ 83,046	$ 63,373	$ 146,419
	Total number of State/LGA/Ward	196	343	529	279	211	490
	Costs per stakeholder’s engagements	$ 323	$ 272	$ 293	$ 298	$ 300	$ 299
	Costs per 1,000 targeted children	$ 65.30	$ 84.90	**$ 52.70**	$ 34.04	$ 120.00	**$ 49.34**
	Number of stakeholder’s engagement per 100,000 children	20	31	18	11	40	17
	Media engagements	$ 15,689	$ 15,615	$ 15,689	$ 15,615	$ 15,615	$ 31,229
	Total number of State	1	1	1	1	1	2
	Costs per media engagement	$ 15,689	$ 15,615	$ 15,689	$ 15,615	$ 15,615	$ 15,615
	Costs per 1,000 targeted children	**$ 16.16**	**$ 14.20**	$ 5.33	$ 6.40	$ 29.57	$ 10.52
	Number of media engagement per 100,000 children	0.10	0.09	0.03	0.04	0.19	0.07
	Pre-Implementation Tracking	$ 514	$ 597	$ 764	$ 597	$ 764	$ 1,361
	Total number of personnel	1	1	1	1	1	2
	Costs per pre-implementation tracking	$ 514	$ 597	$ 764	$ 597	$ 764	$ 680
	Costs per 1,000 targeted children	$ 0.53	$ 0.54	$ 0.26	$ 0.24	$ 1.45	$ 0.46
	Number of pre-implementations tracking per 100,000 children	0.10	0.09	0.03	0.04	0.19	0.07
**Training**							
	Implementation trainings (State/LGA/Ward/Independent Monitors)	$ 11,131	$ 22,738	$ 29,034	$ 40,216	$ 14,977	$ 55,194
	Total number of personnel	1,740	1,371	5,077	1,879	856	2,735
	Costs per personnel trained	$ 6.40	$ 16.58	$ 5.72	$ 21.40	$ 17.50	$ 20.18
	Costs per 1,000 targeted children	$ 11.46	$ 20.68	$ 9.87	$ 16.49	$ 28.36	$ 18.60
	Number of personnel trained per 100,000 children						
	Micro-Plan process (National/State/Ward) (unit)	$ 13,862	$ 11,570	$ 35,400	$ 14,730	$ 9,192	$ 23,922
	Total number of personnel	1385	548	4147	1136	305	1441
	Costs per micro-plan process	$ 10.01	$ 21.11	$ 8.54	$ 12.96	$ 30.13	$ 16.60
	Costs per 1,000 targeted children	**$ 14.28**	**$ 10.52**	**$ 12.04**	$ 6.04	$ 17.41	**$ 8.06**
	Number of micro-plan process per 100,000 children	143	50	141	47	58	49
	Micro-Plan Verification	$ 820	$ 1,138	$ 847	$ 904	$ 1,124	$ 2,028
	Total number of personnel	2	2	3	2	2	4
	Costs per micro-plan verification	$ 409.85	$ 569.07	$ 282.31	$ 451.84	$ 562.13	$ 506.98
	Costs per 1,000 targeted children	$ 0.84	$ 1.04	$ 0.29	$ 0.37	$ 2.13	$ 0.68
	Number of micro-plan verification per 100,000 children	0.21	0.18	0.10	0.08	0.38	0.13
**Technical Assistance**							
	Technical Assistance	$ 18,898	$ 18,898	$ 18,898	$ 18,898	$ 18,898	$ 37,796
	Total number of personnel	1	1	1	1	1	2
	Costs per technical assistance	$ 18,897.76	$ 18,897.76	$ 18,897.76	$ 18,897.76	$ 18,897.76	$ 18,897.76
	Costs per 1,000 targeted children	**$ 19.46**	**$ 17.19**	$ 6.43	$ 7.75	$ 35.78	$ 12.74
	Number of technical assistances per 100,000 children	0.10	0.09	0.03	0.04	0.19	0.07
**Overhead**							
	Office operational costs	$ 139	$ 139	$ 139	$ 139	$ 139	$ 278
	Number of States office	1	1	1	1	1	2
	Costs per office	$ 138.84	$ 138.84	$ 138.84	$ 138.84	$ 138.84	$ 138.84
	Costs per 1,000 targeted children	**$ 0.14**	**$ 0.13**	$ 0.05	$ 0.06	$ 0.26	$ 0.09
	Number of offices per 100,000 children	0.10	0.09	0.03	0.04	0.19	0.07
	**Total costs per State**	$ 133,396	$ 202,544	$ 281,473	$ 244,864	$ 150,487	$ 395,352
	**Costs per 1,000 targeted children**	$ 137.39	$ 184.21	$ 95.71	$ 100.38	$ 284.96	$ 133.23
	**Budget impact Stand-Alone state**	**$ 45,465**		**$ 110,342**			

Source: Operational budget 2019 measles follow-up and meningitis A catch-up campaign

*Costs are related to implementation of measles SIA. Measles target population: 9 to 59 months

In **Bold** positive effect on the cost category of the integrated campaign

Costs are converted from Nigerian naira (NGN) into United States Dollar (USD) using a United Nation (UN) exchange rate of 360.13 NGN for 1 USD (as at October 2019)

The comparison of the actual costs with the budgeted costs for the campaigns has shown no significant budget variances; Calculations presented are based on budgeted costs

LGA = Local Government Area

The costs of the vaccine delivery components of the integration are reflected in Table 4. The budget impact estimated an overall saving of 138,443 USD between Yobe (stand-alone) and Plateau (integrated), and 440,718 USD between Kano (stand-alone) and Kwara coupled with Kaduna (integrated). During the integration, there was optimization of resources/maximized efficiency and cost reductions for vaccination personnel, implementation materials, supplies and team transportation, and pen markers; leading to lower costs per children. The costs per children for vaccination personnel is higher in the stand-alone strategy (0.172 USD in Yobe and 0.171 USD in Kano) than in the integrated approach (0.110 USD in Plateau and 0.104 USD in Kaduna + Kwara). A decrease in the costs of transport and logistics is also observed for all integrated strategies compared with the stand-alone strategy. Whereas the cost per children of waste management in the integrated strategies are 2.41 times higher than those of the stand-alone strategies.

**Table 4:** Vaccine delivery components costs* by vaccination strategy, Nigeria, 2019

Cost categories	Measles target population (9–59 months)	Stand-Alone	Integrated	Stand-Alone	Integrated	Integrated	Integrated
		Yobe	Plateau	Kano	Kaduna	Kwara	Kaduna + Kwara
		970,946	1,099,512	2,940,912	2,439,351	528,102	2,967,453
**Vaccination personnel**							
	Vaccination Personnel	$ 167,436	$ 121,127	$ 501,654	$ 245,237	$ 63,828	$ 309,065
	Total number of vaccination team	1,295	458	3,921	1,016	220	1,236
	Costs per vaccination team	$ 129.33	$ 264.39	$ 127.93	$ 241.28	$ 290.07	$ 249.96
	Costs per 1,000 targeted children	**$ 172.45**	**$ 110.16**	**$ 170.58**	$ 100.53	$ 120.86	**$ 104.15**
	Number of units/vaccination team per 100,000 targeted children	133	42	133	42	42	42
	Transport allowance for collection of payment	$ 23,304	$ 12,474	$ 70,356	$ 26,325	$ 6,184	$ 32,509
	Total number of vaccination team	1,295	458	3,921	1,016	220	1,236
	Costs per vaccination team	$ 18.00	$ 27.23	$ 17.94	$ 25.90	$ 28.10	$ 26.29
	Costs per 1,000 targeted children	**$ 24.00**	**$ 11.34**	**$ 23.92**	$ 10.79	$ 11.71	**$ 10.96**
	Number of vaccination team per 100,000 targeted children	133	42	133	42	42	42
**Supervision**							
	National Supervision	$ 31,150	$ 17,655	$ 75,984	$ 45,839	$ 32,233	$ 78,072
	Total number of supervisors	36	18	90	47	33	80
	Costs per national supervisors	$ 865.27	$ 980.82	$ 844.26	$ 975.30	$ 976.75	$ 975.90
	Costs per 1,000 targeted children	**$ 32.08**	**$ 16.06**	$ 25.8	$ 18.79	$ 61.04	$ 26.31
	Number of supervisors per 100,000 targeted children	0.000037	0.000016	0.000031	0.000019	0.000062	0.000027
**Implementation Materials**							
	Implementation Materials - State Level	$ 539	$ 191	$ 1,633	$ 423	$ 92	$ 515
	Number of vaccination teams	1,295	458	3,921	1,016	220	1,236
	Costs per implementation materials	$ 0.42	$ 0.42	$ 0.42	$ 0.42	$ 0.42	$ 0.42
	Costs per 1,000 targeted children	**$ 0.56**	**$ 0.17**	**$ 0.56**	$ 0.17	$ 0.17	**$ 0.17**
	Number of implementation materials per 100,000 targeted children	133	42	133	42	42	42
**Transport and logistics**							
	Team and supplies transportation	$ 158,413	$ 123,589	$ 464,990	$ 189,351	$ 71,905	$ 261,256
	Number of wards and vaccination teams	1,473	783	4,405	1,271	414	1,685
	Costs per wards and vaccination teams	$ 107.57	$ 157.81	$ 105.55	$ 148.93	$ 173.67	$ 155.01
	Costs per 1,000 targeted children	**$ 163.15**	**$ 112.40**	**$ 158.11**	$ 77.62	$ 136.16	**$ 88.04**
	Number of supplies and team’ transport per 100,000 targeted children	152	71	150	52	78	57
	Waste Management	$ 1,746	$ 4,786	$ 5,288	$ 10,567	$ 2,320	$ 12,887
	Total syringes	1,197,380	1,355,929	3,626,762	3,008,232	651,261	3,659,493
	Costs per syringes	$ 0.0015	$ 0.0035	$ 0.0015	$ 0.0035	$ 0.0036	$ 0.0035
	Costs per 1,000 targeted children	$ 1.80	$ 4.35	$ 1.80	$ 4.33	$ 4.39	$ 4.34
	Number of syringes per 100,000 targeted children	123,321	123,321	123,321	123,321	123,321	123,321
	Pen Markers (unit)	$ 4,334	$ 1,562	$ 13,054	$ 3,427	$ 756	$ 4,183
	Number of vaccination teams	1,295	458	3,921	1,016	220	1,236
	Costs per vaccination teams	$ 3.35	$ 3.41	$ 3.33	$ 3.37	$ 3.44	$ 3.38
	Costs per 1,000 targeted children	**$ 4.46**	**$ 1.42**	**$ 4.44**	$ 1.40	$ 1.43	**$ 1.41**
	Number of vaccination team per 100,000 targeted children	133	42	133	42	42	42
	**Total costs per State**	$ 386,924	$ 281,383	$ 1,132,958	$ 521,170	$ 177,317	$ 698,487
	**Costs per 1,000 targeted children**	**$ 398.50**	**$ 255.92**	**$ 385.24**	$ 213.65	$ 335.76	**$ 235.38**
	**Budget impact Stand-Alone state**	**$ -138,443**		**$ -440,718**			

Source: Operational budget 2019 measles follow-up and meningitis A catch-up campaign

*Costs are related to implementation of measles SIA. Measles target population: 9 to 59 months

In **Bold** positive effect on the cost category of the integrated campaign

Costs are converted from Nigerian naira (NGN) into United States Dollar (USD) using a UN exchange rate of 360.13 NGN for 1 USD (as at October 2019)

The comparison of the actual costs with the budgeted costs for the campaigns has shown no significant budget variances; Calculations presented are based on budgeted costs

The results presented in Table 5 represents costs related to the coverage survey. Significant cost-savings were observed particularly for trainings, fieldwork, and quality assurance measures in integrated versus stand-alone campaign.

**Table 5:** Costs* of post campaign coverage survey by vaccination strategy, Nigeria, 2019

Cost categories	Measles target population (9–59 months)	Stand-Alone	Integrated	Stand-Alone	Integrated	Integrated	Integrated
		Yobe	Plateau	Kano	Kaduna	Kwara	Kaduna + Kwara
		970,946	1,099,512	2,940,912	2,439,351	528,102	2,967,453
**Trainings**							
	Trainings	$ 757	$ 285	$ 1,847	$ 323	$ 276	$ 598
	Total number of personnel	174	191	448	245	177	422
	Costs per personnel	**$ 4.35**	**$ 1.49**	**$ 4.12**	$ 1.32	$ 1.56	**$ 1.42**
	Costs per 1,000 targeted children	**$ 0.78**	**$ 0.26**	**$ 0.63**	$ 0.13	$ 0.52	**$ 0.20**
	Number of personnel trained per 100,000 targeted children	18	17	15	10	34	14
**Coverage survey**							
	Fieldwork and Quality Assurance Measures	$ 3,052	$ 833	$ 7,853	$ 866	$ 730	$ 1,597
	Total number of personnel	157	174	404	222	161	383
	Costs per personnel for fieldwork and quality assurance measures	**$ 19.44**	**$ 4.79**	**$ 19.44**	$ 3.90	$ 4.54	**$ 4.17**
	Costs per 1,000 targeted children	**$ 3.14**	**$ 0.76**	**$ 2.67**	$ 0.36	$ 1.38	**$ 0.54**
	Number of personnel for fieldwork and quality assurance measures per 100,000 targeted children	16	16	14	9	30	13
	Personnel	$ 12,357	$ 18,965	$ 31,142	$ 20,451	$ 16,758	$ 37,209
	Total number of personnel	174	191	448	245	177	422
	Costs per personnel	$ 71.02	$ 99.30	$ 69.51	$ 83.47	$ 94.68	$ 88.17
	Costs per 1,000 targeted children	$ 12.73	$ 17.25	$ 10.59	$ 8.38	$ 31.73	$ 12.54
	Number of personnel per 100,000 targeted children	18	17	15	10	34	14
	**Total costs per State**	$ 16,165	$ 20,084	$ 40,841	$ 21,640	$ 17,764	$ 39,404
	**Costs per 1,000 targeted children**	$ 16.65	$ 18.27	**$ 13.89**	$ 8.87	$ 33.64	**$ 13.28**
	**Budget impact Stand-Alone state**	$ 1,570.33		$ -1,789.41			

Source: Operational budget 2019 measles follow-up and meningitis A catch-up campaign

*Costs are related to implementation of measles SIA. Measles target population: 9 to 59 months

In **Bold** positive effect on the cost category of the integrated campaign

Costs are converted from Nigerian naira (NGN) into United States Dollar (USD) using a UN exchange rate of 360.13 NGN for 1 USD (as at October 2019)

The comparison of the actual costs with the budgeted costs for the campaigns has shown no significant budget variances; Calculations presented are based on budgeted costs

Total number of personnel during training: Training facilitators, supervisors, lead consultant and data collectors

Total number of personnel for fieldwork and quality assurance measures: supervisors, lead consultant and data collectors

Total number of personnel: Data collectors, enumerators, training facilitators, lead consultant and supervisors

The overall budget impact of the integration strategies estimated that savings were up to 420,000 USD; in total, over 200 USD per 1,000 children (0.2 USD per children) were saved in the studied states with an integrated measles vaccination campaign. Savings on the coverage survey components were accrued by lower costs in the integration of trainings, and through reduced field work and quality assurance measures costs.

## Discussion

In this study, we have compared the difference in costs generated by two measles vaccination strategies, the stand-alone measles SIA vaccination and the integration of measles and Men-A vaccination with similar health outcomes. The study results show that the use of an integrated measles vaccination approach implies a greater saving versus a stand-alone measles vaccination approach. Many common activities targeting the same actors were integrated, including but not limited to planning and coordination meetings, training of health workers, advocacy, communication and social mobilization activities, transport of vaccines and other supplies, coverage surveys, etc. Such integration resulted in cost reductions which may be allocated to other priority areas.

It is to note that, the number of vaccination teams were quite high in the stand-alone measles campaign states of Kano and Yobe (3.2 times higher number of teams in Kano as compared to the matched states, and 2.8 times higher in Yobe as compared to Plateau State). This account for a significant proportion of the cost-of-service delivery and may affect the operational costs related to team deployment and implementation.

In low- and middle-income countries, on average, operational costs for SIAs are high [19], and consistently higher in the African region than other WHO regions [21]. Major cost drivers are usually related to human resources/personnel costs and, transport and logistics costs [22]. Campaign planning and preparation components appear costlier in the integrated measles vaccination strategy as integrating injectable during SIA require higher engagement with the communities and an increasing production and distribution of information, education, and communication materials. Additionally, more trainings and job aids for the health workers were needed to ensure proper administration, handling, and recording of vaccines.

Among lessons learned in the 2019 integration were better team distributions regarding terrain, population density and consideration of workload estimates in microplanning. The number of teams per state is calculated based on a standard workload of a vaccination team (number of vaccinations per campaign period) and the size of the target population per state (number of teams = target population/workload). The team composition varies with stand-alone campaign requiring 2 vaccinators and 2 recorders per team while the integrated campaign require 3 vaccinators and 3 recorders per team. However, the integrated campaign delegated the team supervision function to the most senior vaccinator while the stand-alone strategy required a separate supervisor as member of the team. As the workload was higher during the integration, a lower number of vaccination team was needed. By contrast, more resources were needed for waste management in the measles integrated strategy, as more waste was generated when integrating two injectable vaccines.

Several studies on the costs and effects of SIAs showed that most interventions (e.g., mass campaigns, accelerated immunization campaign, community education) increased the coverage of vaccinated children, and reported positive cost-effectiveness ratios [23]. A study by Colombini et al. found that the introduction and sustained use of MenAfriVac® to prevent Men-A has a substantial positive economic impact, both for the health system and for households [24]. In addition, a cost-effectiveness study of SIA for measles in Uganda found that the addition of SIAs made outbreaks less frequent and lower in magnitude [25]. Whatever the strategy used, measles immunization is known to be a cost-effective intervention [26] comparable to our results on cost reductions of an integrated campaign. The integration provides opportunities for future improvement with the time gained by offering one-stop services at the point of delivery and, the potential further reduction of missed opportunities for vaccination and in the burden of vaccine preventable diseases with higher immunization coverage.

This study is the first assessment of the economic impact of measles vaccination strategy with Men-A vaccines or other injectable vaccines (e.g., Yellow Fever, Polio inactivated, Human papillomavirus, tetanus etc.). The strength of our analysis is that we used the verified target population for the cost per children and vaccination-related costs. The last population census was conducted in Nigeria in 2006 and, the verification micro-plan process provided a more realistic number of eligible children present at administrative levels. Another strength is that the variance between budgeted and actual expenditures is not significant. This indicate that all activities that were planned took place, and that campaigns objectives were on track.

This study has some limitations, including the lack of data to assess additional costs from Government counter-part funding. Moreover, our study did not look at the costs for reaching hard-to-reach populations, and previously unvaccinated (zero dose) children. We note that variations exist in the cost per activity across different states, indicating the need for accommodating the tailoring of funding of vaccination activities based on the micro-plans and the realities on the ground.

This study provides decision-makers with a framework for allocating resources to determine financial costs for structured planning to effectively roll-out an integrated vaccination campaign. The COVID-19 situation offers new opportunities and need for integration across immunization programmes, which would be in line with our study findings to save costs with integrated immunization programmes. However, it also imposes new considerations such as personal protection equipment requirements for vaccination personnel which will potentially increase the cost of vaccine delivery.

We conclude that in the state settings evaluated in this study, the more efficient team composition and workload management during vaccination as well as foregoing some coverage survey activities for the integrated measles-Men-A campaign offset its higher planning costs relative to the stand-alone measles campaign, considering only operational costs funded primarily by Gavi and the Federal Government of Nigeria. This can be expected to translate to greater value in improving access and efficiency as integration of programmes, through sharing of costs, makes more life-saving interventions accessible to communities [6]. The cost to reach zero dose children would be worthwhile to estimate in the future.

## Data Availability

Data used during the study (e.g., the financial and technical reports) are available from the authors on reasonable request. Data for the post campaign coverage survey are also available and can be obtain on request from the National Bureau of Statistics (NBS) in Nigeria.

## Abbreviations

ACSM: Advocacy, Communication and Social Mobilization
AEFI: Adverse Event Following Immunization
DIPs: Daily Implementation Plans
FCT: Federal Capital Territory
LGAs: Local government areas
NPHCDA: National Primary Health Care Development Agency
SIA: Supplemental immunization activities
WHO: World Health Organization
